# Intensive care unit occupancy predictions in the COVID-19 pandemic based on age-structured modelling and differential flatness

**DOI:** 10.1007/s11071-022-07267-z

**Published:** 2022-02-18

**Authors:** Christoph Hametner, Lukas Böhler, Martin Kozek, Johanna Bartlechner, Oliver Ecker, Zhang Peng Du, Robert Kölbl, Michael Bergmann, Thomas Bachleitner-Hofmann, Stefan Jakubek

**Affiliations:** 1 Institute of Mechanics and Mechatronics, TU Wien, Getreidemarkt 9, 1060 Vienna, Austria; 2 Division of Visceral Surgery, Department of General Surgery, Medical University of Vienna, Vienna, Austria; 3 Comprehensive Cancer Center, Medical University of Vienna, Vienna, Austria

**Keywords:** SARS-CoV2, COVID-19, Epidemiological modelling, Differential flatness, Dynamical systems

## Abstract

The COVID-19 pandemic confronts governments and their health systems with great challenges for disease management. In many countries, hospitalization and in particular ICU occupancy is the primary measure for policy makers to decide on possible non-pharmaceutical interventions. In this paper a combined methodology for the prediction of COVID-19 case numbers, case-specific hospitalization and ICU admission rates as well as hospital and ICU occupancies is proposed. To this end, we employ differential flatness to provide estimates of the states of an epidemiological compartmental model and estimates of the unknown exogenous inputs driving its nonlinear dynamics. A main advantage of this method is that it requires the reported infection cases as the only data source. As vaccination rates and case-specific ICU rates are both strongly age-dependent, specifically an age-structured compartmental model is proposed to estimate and predict the spread of the epidemic across different age groups. By utilizing these predictions, case-specific hospitalization and case-specific ICU rates are subsequently estimated using deconvolution techniques. In an analysis of various countries we demonstrate how the methodology is able to produce real-time state estimates and hospital/ICU occupancy predictions for several weeks thus providing a sound basis for policy makers.

## Introduction

The ongoing fight against recurring epidemic waves of the SARS-CoV-2 pandemic is a complex undertaking that requires several key directions of action such as: (i) social distancing and personal protective equipment, (ii) testing for infection, (iii) quarantine of infected people and (iv) vaccination.

Management of healthcare systems which are treating the actual COVID-19 patients remains a central issue during the ongoing pandemic. The necessity to avoid overloading of the healthcare system is imperative as the level and quality of medical care and particularly mortality are directly related to the available capacities. Within the healthcare system, the ICU capacity constitutes a major bottleneck for policymakers to take appropriate measures.

Therefore, predictions of admissions to hospitals and intensive care units (ICUs) are a critical factor in the decision-making process. They are equally important in the phases of upcoming surges and in phases of decline, where far-reaching measures such as lockdowns should be lifted as quickly as possible.

### Occupancy models

The dynamics of the epidemic along with the time lag between the infection and the actual hospitalization as well as the duration of stay (ranging from a few days to several weeks) make it difficult to predict the occupancy of COVID-19 patients in hospitals and ICUs in particular.

To infer future hospital and ICU occupancies from reported case numbers, in this paper an approach that comprises the online estimation of case-specific hospitalization/ ICU admission rates is proposed using deconvolution techniques. It explicitly accounts for time lags between infection and hospitalization as well as for the distribution of the length of stay. To allow for this approach to make predictions depending on different future evolutions of infections, an age-structured compartmental model is proposed along with a methodology that provides estimates of the states of that model and the otherwise unknown exogenous inputs driving its dynamics requiring only the reported infection data.

### Modelling approach and review

The compartmental model utilized in this work is based on the classical SIR-model [1] but has been extended recently by an additional compartment (’contact-less’) in combination with an approach to determine unknown exogenous input *u*(*t*) driving its dynamics [2]. The latter is based on the concept of differential flatness [3, 4]. The exogenous input *u*(*t*) aggregates all unknown drivers of the epidemic and thus enables the realistic analysis and prediction of epidemiological dynamics, in particular recurring waves.

The methodology in [2] is now further augmented by segregation into two coupled age groups in order to account for the fact that both hospitalization and ICU occupancy are strongly age-dependent. Age-structured compartmental models are commonly used in epidemiology. For example, the recent publication [5] proposes two age groups to model the spread of tuberculosis. A more complex stochastic compartmental model of the COVID-19 pandemic is utilized in [6] where five age groups are employed. Utilizing data for Belgium they also predict hospitalization and ICU occupancy. No estimation of external drivers of the epidemic is given, though, and the prediction accuracy for new hospitalizations can hardly be determined based on the presented validation. Data from the COVID-19 pandemic in Switzerland is treated in [7]. A discrete model based on compartments with explicit duration of the individual phases is presented. Seventeen age groups are distinguished, and both hospitalization and ICU occupancy for these groups are computed. The model is parametrized with the measured age distribution in the hospitals. Although several simulations are shown to analyze the effects of different measures, no methodology for real-time predictions is presented. A compartmental model with four age groups is presented in [8] which focuses on an optimal control approach and presents simulations of closed-loop scenarios.

The topology of the population is also an important factor to impact the epidemics. In [9] an extended SIR model is presented which describes the investigation of the epidemic spread in complex networks by considering the propagation vector and infection delays. In this context, [10] studies the co-evolution of multiple information dissemination and epidemic under the influence of mass media.

A Monte-Carlo SEIR model is employed in [11] to model and simulate COVID-19 outbreaks in the Netherlands, South Korea, China and Italy. Although the model is used for forecasts of case numbers and hospitalizations, ICU occupancy is not treated but the forecasts could not be validated. In [12] a SEIAR model is presented with four distinct age groups for the COVID-19 pandemic in Hunan and Jilin provinces. The model was parameterized based on literature and own data fitting, respectively. The main result is the estimation of SAR-values (secondary attack-rate values) between different age groups.

In [13] a methodology to forecast the patient numbers admitted to hospital and ICU is presented. The model is restricted to data from the hospital and no generic pandemic model is included, but time-varying admission rates are estimated. Forecasts up to five days as well as the respective validation data are presented. In [14] the hospitalization rate is estimated for New Mexico by pre-filtering and dividing reported hospitalization cases by reported COVID-19 case numbers; no pandemic model is employed. Several different models are compared and analyzed in [15] for forecasting ICU occupancy. The model outputs are merged by a trimmed-mean approach to predict the occupancy directly. Predictions and validation data is presented for two-week forecasts, however, age segregation is not considered. Another model without age segregation is presented in [16]. ICU admission rates, time lag until ICU admission and duration of intensive care are directly estimated. Predictions show squared correlation values between 0.00 and 0.99 depending on the data set, the better values being achieved during exponential growth phases. Data from a publicly available forecasting tool [17] are processed to obtain statistical estimates of occupancy. This statistical model is then trained to minimize the prediction error. No age segregation is considered and especially during peak values of the pandemic model deviations are considerable. The trimmed mean of autoregressive, machine learning, and epidemiological models is utilized in [15] for estimating the ICU occupancy. One- and two-week predictions are shown together with reported cases, but no distinction is made between age groups.

A different approach is shown in [18], using static and dynamic incidence models as well as a care pathway model to generate predictions for ICU and hospital beds. Using local data from health districts in Germany, forecasts were made several weeks into the future. However, these were compared to the actual cases only for the first 23 days, mostly falling outside the 95% confidence interval. A differentiation of the hospital data into age groups was also not considered here. A prediction of hospital and ICU beds was also performed by [19], using an adjusted SEIRD model for its determination. A two-week forecast was shown for the Mexico City metropolitan area, which was also compared to actual cases. However, no predictions were shown at other time periods, making it difficult to draw conclusions about the performance of the model. In addition, no age classification was considered.

Besides the hospitalization numbers, the *case-specific* hospitalization and *case-specific ICU admission rates* $r_\text{ H }$ and $r_\text{ ICU }$, respectively, constitute decisive latent variables in the analysis of the epidemic. In the method presented in this paper both case-specific admission rates are treated as variable over time and a methodology is proposed to estimate them in real-time using deconvolution techniques. To this end, only the reported active cases along with hospital and ICU occupancy data are required. Another approach to model ICU occupancy is presented in [16]. However, the case-specific admission rates are not estimated by deconvolution but are directly taken from literature or estimated externally.

### Methodology

**Fig. 1:**
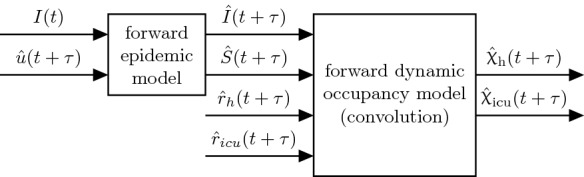
Schematics of the proposed methodology to estimate and predict the occupancies $\hat{{\upchi }}$ for hospital and ICU

In the methodology proposed in this work, the predictions of hospital and ICU occupancy, respectively, are based on the predictions of active COVID-19 case numbers using the flatness approach [2] along with projections of the case-specific admission rates, cf. Fig. 1. Predictions of hospital and ICU occupancies $\hat{{\upchi }}_\text{ h }$ and $\hat{{\upchi }}_\text{ icu }$ are obtained from a *forward dynamic occupancy model*, which by itself uses predictions of the states of an *forward epidemic model* (i.e. the number of infected $\hat{I}$, susceptibles $\hat{S}$) and case-specific admission rates $\hat{r}_\text{ h,icu }$.

To this end, in Sect. 2, an age-structured compartmental epidemic model is introduced and it is shown how the unknown exogenous inputs *u* that drive its dynamics can be estimated. Subsequently, it is demonstrated how these inputs are utilised to obtain predictions of the future epidemiological states $\hat{I}(t+\tau )$ and $\hat{S}(t+\tau )$ using only information available up until time *t*. These predictions are then used for the occupancy predictions $\hat{{\upchi }}(t+\tau )$.

In the proposed approach the case-specific hospitalization and ICU admission rates $\hat{r}_\text{ h }$ and $\hat{r}_\text{ icu }$ are treated as unknown latent variables. Besides the infection dynamics itself, they are the second major factor that seriously impacts the burden on ICUs and hospitals. In Sect. 3 it is described how these admission rates can be estimated in real-time based on deconvolution techniques.

Finally, dynamic occupancy forecasts $\hat{{\upchi }}(t+\tau )$ for different countries are presented and discussed in Sect. 4.

## Age-structured compartmental model with exogenous drivers

The description of epidemics is often based on compartmental models [20] which are versatile approaches and can easily be extended or adapted, as shown in [21–23]. In this section, the compartmental model with exogenous drivers as presented in [2] is recaptured and then augmented to a discrete, age-structured model. The estimates of the epidemiological states obtained from the proposed segregated modelling approach provide the basis for estimating the hospital and ICU occupancy.

### Compartmental models with exogenous drivers

Compartmental epidemiological models constitute a set of coupled autonomous ordinary differential equations. The dynamics are exclusively determined by the initial states and the parameters. However, the COVID-19 pandemic is significantly driven by exogenous inputs. There are different approaches to extend compartmental models to non-autonomous systems with exogenous inputs, e.g. [24–26], so that they can accurately describe multiple epidemic waves. In this work, we focus on the methodology introduced in [2] which is based on the mathematical property of differential flatness.

Various compartmental models have the property of differential flatness [27, 28]. Extending them with exogenous drivers *u*(*t*) [2] is briefly discussed for the SIR model:

1 $$\begin{aligned} \left\{ \begin{array}{l} \dot{S} = - \frac{\beta I S}{N} + u(t) \\ \dot{I} = \frac{\beta I S}{N} -\gamma I \\ \dot{R} = \gamma I, \end{array}\right. \end{aligned}$$

with *I* as the number of infected or active cases, *S* as the susceptibles, *R* as the recovered individuals and *N* as the population size. The transmission rate $\beta$ and the recovery rate $\gamma$ are parameters of the system. The exogenous driver *u*(*t*) can be obtained by differentiating system (1) with respect to time. Due to the extension of system (1) with exogenous drivers, the assumption that *N* is the sum of compartments *S*, *I*, *R* does not hold. Therefore, the additional compartment of *contact-less* *C* is introduced. The population size is thus

$$\begin{aligned} N = C + S + I + R, \end{aligned}$$

where *C* has no additional state equation. Differential flatness entails that *u*(*t*) and *S*(*t*) can be expressed solely by *I*(*t*) and a finite number of its derivatives $\dot{I}$ and $\ddot{I}$, as will be shown for an age segregated CSIR model in the sequel.

### Age-structured CSIR compartmental model

In order to account for how much more severely COVID-19 affects the elderly, it is appropriate to segregate the CSIR model into two discrete but coupled age groups, indicated by “1” and “2”:

2 $$\begin{aligned} \dot{S}_1&= -\left( \frac{2\beta I_{1}}{N_{1}-1}+\frac{\beta _{12}I_{2}}{N_{2}}\right) S_{1} + {u}_{1} \end{aligned}$$

3 $$\begin{aligned} \dot{S}_2&= -\left( \frac{2\beta I_{2}}{N_{2}-1}+\frac{\beta _{21}I_{1}}{N_{1}}\right) S_{2} + {u}_{2} \end{aligned}$$

4 $$\begin{aligned} \dot{I}_1&= \left( \frac{2\beta I_{1}}{N_{1}-1}+\frac{\beta _{12}I_{2}}{N_{2}}\right) S_{1} - \gamma I_{1} \end{aligned}$$

5 $$\begin{aligned} \dot{I}_2&= \left( \frac{2\beta I_{2}}{N_{2}-1}+\frac{\beta _{21}I_{1}}{N_{1}}\right) S_{2} - \gamma I_{2} \end{aligned}$$

6 $$\begin{aligned} \dot{R}_1&= \gamma I_1 \end{aligned}$$

7 $$\begin{aligned} \dot{R}_2&= \gamma I_2 \end{aligned}$$

In Equations (2–7), the compartments *S*, *I* and *R* are split into the two age groups. The age limit for the segregation into these groups is 65 years for Austria but can vary depending on the investigated country and the available data.

**Fig. 2:**
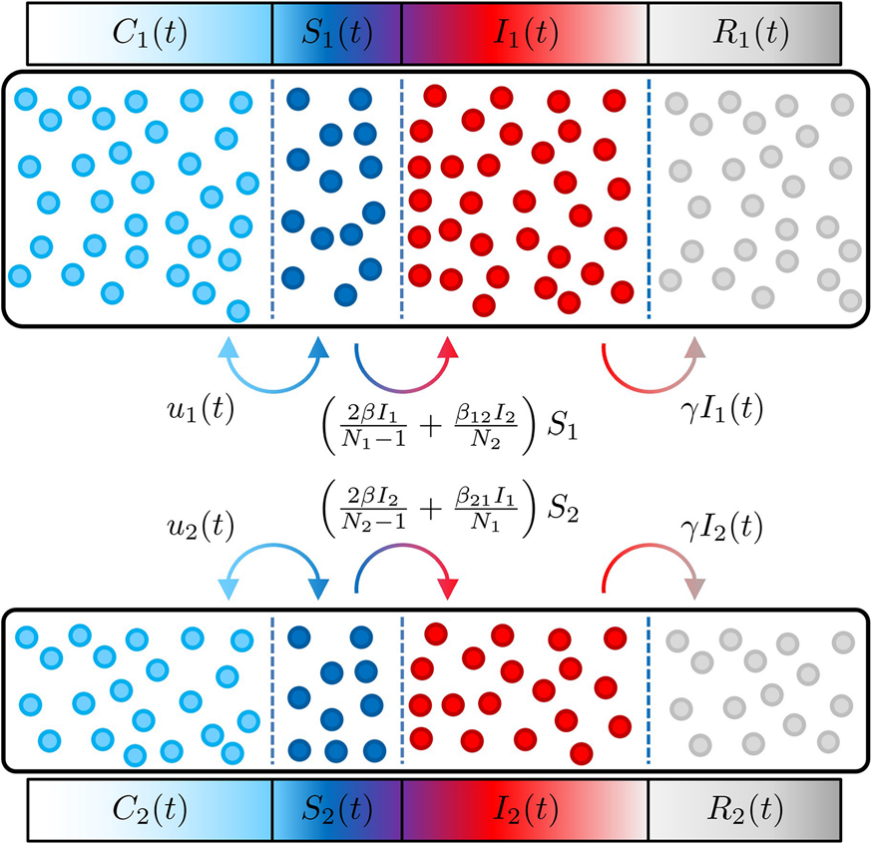
Age-structured CSIR compartmental model showing the flow of individuals between the compartments and the couplings between the age groups

Equations (2–7) are visualised in Fig. 2, where the flows of individuals between the compartments and their couplings, including compartments $C_1$ and $C_2$, are shown. Notably, each age group is driven by its own exogenous input $u_{1,2}$ making it a multi-input system.

The system represented by (2–7) utilises the three transmission rates $\beta , \beta _{12}$ and $\beta _{21}$ which describe transmissions within and between the age groups. The transmission rates and the recovery rate $\gamma$ constitute the parameters of the compartmental model.

For the estimation of the ICU and hospital occupancy presented in the following sections, age group specific production rates are introduced:

8 $$\begin{aligned} \begin{aligned} \pi _1(t)&= \lambda _1(t) S_1(t) \\ \pi _2(t)&= \lambda _2(t) S_2(t) \end{aligned} \end{aligned}$$

Here, $\lambda _{1,2}$ represent the forces of infection for the specific age groups and $S_{1,2}$ the susceptibles, respectively. Based on equations (2-5), they are given by

9 $$\begin{aligned} \begin{aligned} \lambda _1&= \left( \frac{2\beta I_{1}}{N_{1}-1}+\frac{\beta _{12}I_{2}}{N_{2}}\right) \\ \lambda _2&= \left( \frac{2\beta I_{2}}{N_{2}-1}+\frac{\beta _{21}I_{1}}{N_{1}}\right) \end{aligned} \end{aligned}$$

which establishes a cross-coupling of the dynamics of the two age groups.

### Estimation of unknown exogenous drivers

Much like the basic SIR model (1) the age segregated model has the property of differential flatness, differing only in the aspect that now a multi-input multi-output system is considered [29, 30]. Specifically, using the reported case numbers $I_1(t)$ and $I_2(t)$ and their first and second derivatives, respectively, one can derive algebraic equations for the number of susceptibles $S_{1,2}$:

10 $$\begin{aligned} \begin{aligned} S_1 = \frac{\dot{I}_1 + \gamma I_1}{\frac{2\beta I_1}{N_1 -1} + \frac{\beta _{12}I_2}{N_2}} \\ S_2 = \frac{\dot{I}_2 + \gamma I_2}{\frac{2\beta I_2}{N_2 -1} + \frac{\beta _{21}I_1}{N_1}} \\ \end{aligned} \end{aligned}$$

The exogenous drivers $u_1$ and $u_2$ are obtained by deriving equations (4-5) with respect to time. After replacing $\dot{S}_{1,2}$ with equations (2-3) and $S_{1,2}$ with (10), the exogenous drivers $u_{1,2}$ for the discrete age groups are obtained as:

11 $$\begin{aligned} u_1= & {} (\dot{I}_1 + \gamma I_1) + \frac{\ddot{I}_1 + \gamma \dot{I}_1}{\frac{2\beta I_1}{N_1 -1} + \frac{\beta _{12}I_2}{N_2}}- \nonumber \\&- \frac{(\dot{I}_1+\gamma I_1) \left( \frac{2\beta \dot{I}_1}{N_1 -1}+\frac{\beta _{12} \dot{I}_2}{N_2}\right) }{\left( \frac{2\beta I_{1}}{N_{1}-1}+\frac{\beta _{12}I_{2}}{N_{2}}\right) ^2} \nonumber \\ u_2= & {} (\dot{I}_2 + \gamma I_2) + \frac{\ddot{I}_2 + \gamma \dot{I}_2}{\frac{2\beta I_2}{N_2 -1} + \frac{\beta _{21}I_1}{N_1}} - \nonumber \\&- \frac{(\dot{I}_2+\gamma I_2) \left( \frac{2\beta \dot{I}_2}{N_2 -1}+\frac{\beta _{21} \dot{I}_1}{N_1}\right) }{\left( \frac{2\beta I_{2}}{N_{2}-1}+\frac{\beta _{21}I_{1}}{N_{1}}\right) ^2} \end{aligned}$$

**Fig. 3:**
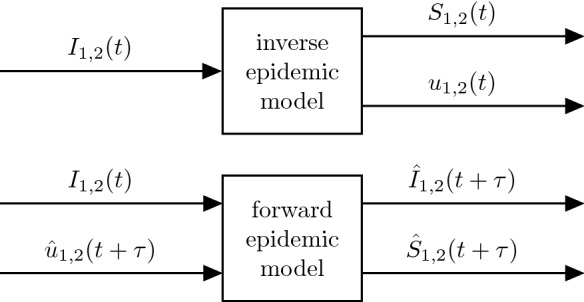
Top: Using the differential flatness property, an inverse use of the epidemic model provides real-time estimates of its states as well as its exogenous inputs that drive the dynamics. Bottom: Using projections of the exogenous inputs, the compartmental epidemic model provides predictions of its future states

Using the time course of $u_1(t)$ and $u_2(t)$ determined from (11), one could reproduce the observed dynamic behaviour of the epidemic for each age group *exactly*. In this sense, (11) can be interpreted as the *inverse* of the epidemic model, Fig. 3 (top). How $u_1(t)$ and $u_2(t)$ can be used to describe and forecast the pandemic is addressed in the following section.

### Analysis and forecasts based on exogenous drivers

A real-time analysis of the COVID-19 epidemic of a country involves the determination of its missing states $S_{1,2}(t)$ as well as its exogenous drivers $u_{1,2}(t)$ using the equations outlined in the previous section. Conceptually this can be interpreted as an inversion of the age segregated *inverse epidemic model*, Fig. 3 (top).

Once the current states of the model are available, its future state trajectory is solely defined by the future course of its exogenous drivers (e.g. $\hat{u}(t+\tau )$). Hence, forecasts of the epidemic can be obtained by suitable projections of $\hat{u}_{1,2}(t+\tau )$) which are then fed into the epidemic model. This amounts to a *forward* use of the model, Fig. 3 (bottom). The projections $\hat{u}(t+\tau )$ therefore constitute the inputs to predict $\hat{I}(t+\tau )$ and $\hat{S}(t+\tau )$ based on Equations (2–5).

**Fig. 4:**
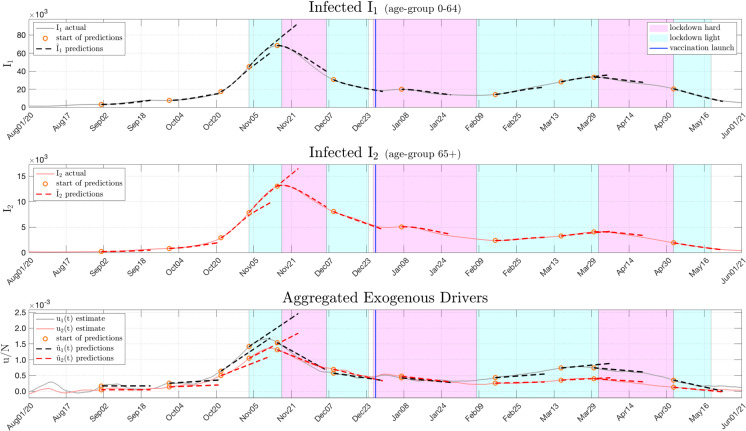
Predicting the course of the disease for Austria. For different points in time, linear projections $\hat{u}_{1,2}$ (dashed lines) of the aggregated exogenous drivers are used to create forecasts of the number of infected three weeks into the future. The actual numbers of infected are based on the cases reported by the Austrian government [31]

Obviously, many different methods could be considered how to obtain the necessary projections of $\hat{u}(t+\tau )$, e.g. regression models or expert knowledge. One suitable approach that resulted in sufficiently accurate projections throughout the pandemic is to apply gradient based linear extrapolations for $\hat{u}$. The strength of this relatively simple approach is its straightforward adaptability to already observable trends in *u*(*t*) and they proved to be a robust and especially transparent method. This is illustrated in Fig. 4 by means of rolling three-week forecasts (i.e. $0<\tau \le 21$) for the active case numbers for Austria. The first age group corresponds to the younger population, aged below 65 years, whereas the second group is related to the elderly at or above 65.

The first two subplots of Fig. 4 show the reported numbers of infected for the two age groups $I_{1,2}$ (solid lines). These are to be compared against the rolling forecasts $\hat{I}_{1,2}(t+\tau )$ (dashed lines) for different points in time. The beginning of each forecast is marked by a circle. The estimated aggregated exogenous drivers $u_{1,2}(t)$ obtained from the inverse epidemic model are displayed as solid lines at the bottom of Fig. 4, together with the linear projections of $\hat{u}_{1,2}(t+\tau )$ (dashed) which are required to obtain the case number forecasts. It can be observed, that the actual courses of the infected are predicted accurately throughout each three-week prediction phase for both age groups in most cases. Notably, a correct prognosis in the vicinity of the peak in fall 2020 in Austria proves to be difficult as the prediction range contained an unpredicted lockdown. The corresponding results for France and the Netherlands are given in the Appendix in Figs. 11 and 15, respectively.

## Estimation of case-specific hospitalization and case-specific ICU admission rates for age groups

In addition to the number of infected people, a key indicator for the severity of the epidemic situation of a country are the remaining capacities of hospital and ICU beds. The methodology to predict the occupancies is based on the case-specific hospitalization/ ICU admission rates. They are defined as the share of patients who are e.g. admitted to ICU following an infection.

While in many related works case-specific admission rates are treated as given and constant over time [16, 32, 33], we propose an approach where these rates are time variant and hence have to be determined from the available data. Especially in view of the upcoming phase of COVID-19 where vaccination rates in many countries are on a steady increase, case-specific admission rates serve as important latent variables that can give valuable hints about the success of vaccination campaigns.

### Hospitalization and ICU occupancy model

An important aspect for modelling and estimating the occupancies is the available data. Particularly important is data that links the length of stay durations in hospital and ICU care to the day of admission. For age-structured models, this information needs to be reported in an even higher resolution. If these data are available, convolution based approaches to model the occupancies can be considered [16].

To this end we define $\varphi (t-\theta )$ as the conditional probability of being in care on a certain day *t*, conditional to prior admission a number of days ($t-\theta$) earlier. Using the available hospitalization statistics, $\varphi (t-\theta )$ can be determined in a straightforward way. Similar to [16], convolution techniques are used to link the current production rate of infections $\pi (t)$ in (8), the conditional probabilities $\varphi (t-\theta )$ as well as the associated case-specific admission rates *r*(*t*) to determine occupancies.

The approach to determine the occupancies $$\upchi $$$$_\text {a}$$ for the two age groups *a*=1,2 resulting from the above considerations is expressed by the discrete convolution

12 $$\begin{aligned} \begin{aligned} {{\upchi }}_\text {h,a}(t)&= \sum \limits _{\theta =t-d_\text {max}}^t \varphi _\text {h,a}(t-\theta ) r_\text {h,a}(\theta ) \pi _\text {a} (\theta ) \\ {\upchi }_\text {icu,a}(t)&= \sum \limits _{\theta =t-d_\text {max}}^t \varphi _\text {icu,a}(t-\theta ) r_\text {icu,a}(\theta ) \pi _\text {a} (\theta ) \end{aligned} \end{aligned}$$

for hospital beds (“h”) and intensive care units (“icu”), respectively. In (12), $\pi _\text {a}$ is the number of new infection cases of the respective age group according to (8), $r_\text {h,a}$ and $r_\text {icu,a}$ are the time varying case-specific hospitalization and case-specific ICU rates. The maximum number of days individuals are considered to be in care is denoted by $d_\text {max}$.

### Estimation of case-specific rates based on deconvolution

**Fig. 5:**
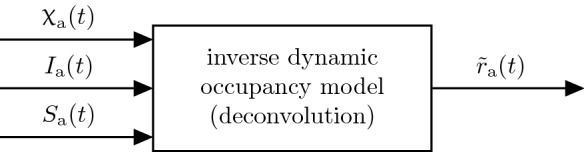
The case and age specific admission rates $\tilde{r}_\text{ a }$ are estimated in real-time using a dynamic occupancy model in combination with deconvolution techniques. Therein, the states of the epidemic model (i.e. $I_\text {a},S_\text {a}$) and the reported occupancies ${\upchi }_\text{ a }$ are utilized

**Fig. 6:**
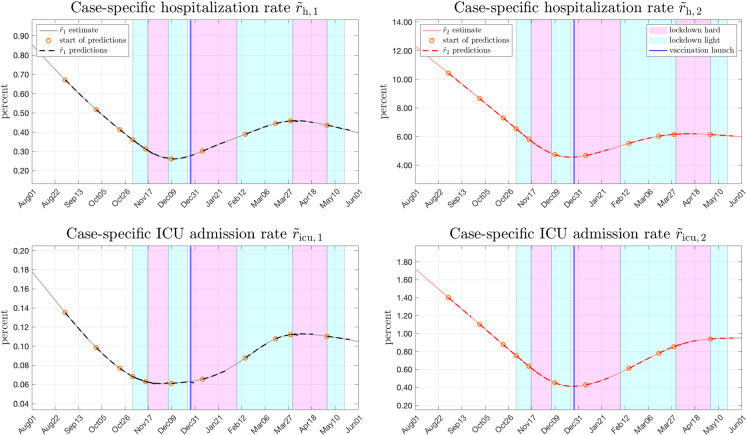
Case-specific hospitalization and ICU admission rates in Austria for both age groups (solid lines), including rolling forecasts (dashed lines). A case-specific admission rate of 100% means, that every infected person has been hospitalized as well. The forecasts $\hat{r}$ start on the same days as the projections of $\hat{u}$ in Fig. 4

In (12), $r_\text {a}$ can be seen as a latent variable which has an important meaning for the pandemic: As will be shown later (cf. Fig. 6) the case-specific admission rates show significant fluctuations over time. This can be due to e.g. vaccination effects or new trends in medical treatment policies. In this work, the case-specific admission rates are therefore treated as time-varying and are estimated based on available measurements as presented schematically in Fig. 5.

Much like the exogenous inputs that drive the epidemic, estimation of the case-specific admission rates is based on the principle of dynamic inversion, whereas this time specifically the occupancy model is considered. Based on the epidemiological states $I_\text {a}(t),$ $S_\text {a}(t)$ from (2-5) and the reported occupancies (e.g. ${\upchi }_\text{ h,a }(t)$) estimates of the case-specific admission rates $\tilde{r}_\text{ a }(t)$ are obtained using deconvolution techniques. These are also known under the names *polynomial division* or *backsolving* [34–36]. For this matter, a Toeplitz matrix ${\varvec{\Psi }}$ is introduced which contains the conditional probabilities $\varphi (t)$ as

$$\begin{aligned} {\varvec{\Psi }} = \left[ \begin{matrix} \varphi (1) &{} 0 &{} 0 &{} \ldots \\ \varphi (2) &{} \varphi (1) &{} 0 &{} \ldots \\ \varphi (3) &{} \varphi (2) &{} \varphi (1) &{} \ldots \\ \vdots &{} \vdots &{} \vdots &{} \vdots \\ \varphi (d_\text {max}) &{} \varphi (d_\text {max}-1) &{} \varphi (d_\text {max}-2) &{} \ldots \\ 0 &{} \varphi (d_\text {max}) &{} \varphi (d_\text {max}-1) &{} \ldots \\ 0 &{} 0 &{} \varphi (d_\text {max}) &{} \ldots \\ \vdots &{} \vdots &{} \vdots &{} \ddots \end{matrix} \right] \end{aligned}$$

for each respective age group^1^. Second, an $n \times 1$ vector $\mathbf {p}$ containing the production rates $\pi (t_j)$ at each observation time $t_j$ ($j=1,\ldots ,n$) is defined as

$$\begin{aligned} \mathbf {p} = \begin{bmatrix} \pi (1)&\pi (2)&\pi (3)&\ldots&\pi (n-2)&\pi (n-1)&\pi (n) \end{bmatrix}^\text {T}. \end{aligned}$$

The last observation at $t_n$ might be the day the estimation of *r* itself is conducted, if data are available up to this day. In the same fashion the $n \times 1$ vector of observed occupancies

$$\begin{aligned} \mathbf {x} = \begin{bmatrix} {\upchi }(1)&{\upchi }(2)&{\upchi }(3)&\ldots&{\upchi }(n-2)&{\upchi }(n-1)&{\upchi }(n) \end{bmatrix}^\text {T} \end{aligned}$$

and the yet unknown vector of case-specific admission rates $\mathbf {r}$

$$\begin{aligned} \mathbf {r} = \begin{bmatrix} r(1)&r(2)&r(3)&\ldots&r(n-2)&r(n-1)&r(n) \end{bmatrix}^\text {T} \end{aligned}$$

are introduced. Finally, a diagonal matrix ${\varvec{\Phi }}$ is defined as

13 $$\begin{aligned} {\varvec{\Phi }} = \text {diag}({\varvec{\Psi }}\mathbf {p}). \end{aligned}$$

Using the above definitions, under ideal conditions the observed occupancies are formally related to the respective case-specific admission rates through

14 $$\begin{aligned} \mathbf {x} = {\varvec{\Phi }} \mathbf {r}, \end{aligned}$$

which is the equivalent of (12), evaluated for all observed time instants and expressed in vector-matrix notation. There are, of course, several ways to determine an estimate $\tilde{\mathbf {r}}$ of the vector of case-specific admission rates, whereby each of them is inherently linked to the minimization of some kind of error criterion, like, e.g.

15 $$\begin{aligned} \tilde{\mathbf {r}}^* = \min _{\mathbf {r}} \Vert {\varvec{\Phi }} \mathbf {r} -\mathbf {x} \Vert _2^2. \end{aligned}$$

When practically computing $\tilde{\mathbf {r}}$ from reported data it turns out that for several countries the result can be sensitive to fluctuations which stem, e.g. from irregularities in the data reporting process. To overcome this problem, regularisation of the second time derivative of *r*(*t*) proved to be a suitable remedy. The motive for this lies in the nature of the time evolution of admission rates: Even though they are time variant their fluctuation over time and in particular their second derivatives can be associated with, e.g. large scale changes in treatment policies applied in hospitals or with the severity of the cases. As each of these underlying trends is subject to certain smoothness, it is reasonable to introduce regularisation. Then, $\tilde{\mathbf {r}}$ is obtained from

16 $$\begin{aligned} \tilde{\mathbf {r}}^* = \min _{\mathbf {r}} \left\{ \Vert {\varvec{\Phi }} \mathbf {r} -\mathbf {x} \Vert _2^2 + \Vert \mathbf {H} \mathbf {r} \Vert _{\mathbf {Q}}^2 \right\} , \end{aligned}$$

where the matrix $\mathbf {H}$ is chosen such that the regularisation term penalises a measure corresponding to the second time derivative of $\tilde{\mathbf {r}}$. A proper choice is

$$\begin{aligned} \mathbf {H} = \left[ \begin{matrix} 1 &{}\quad -2 &{}\quad 1 &{}\quad 0 &{}\quad \ldots &{}\quad 0 &{}\quad 0 \\ 0 &{}\quad 1 &{}\quad -2 &{}\quad 1 &{}\quad \ldots &{}\quad 0 &{}\quad 0\\ \vdots &{} &{} &{}\quad \ddots &{} &{} &{}\quad \vdots \\ 0 &{}\quad \ldots &{}\quad 0 &{}\quad 1 &{}\quad -2 &{}\quad 1 &{}\quad 0 \\ 0 &{}\quad 0 &{}\quad \ldots &{}\quad 0 &{}\quad 1 &{}\quad -2 &{}\quad 1 \end{matrix} \right] . \end{aligned}$$

Finally, $\mathbf {Q}$ is a diagonal weighting matrix that controls the amount of regularisation.

The solution of (16) can be obtained analytically, yielding

17 $$\begin{aligned} \tilde{\mathbf {r}^*} = \left( {\varvec{\Phi }}^\text {T} {\varvec{\Phi }} + \mathbf {H}^\text {T}\mathbf {Q}\mathbf {H}\right) ^{-1} {\varvec{\Phi }}^\text {T} \mathbf {x} \end{aligned}$$

Equation (17) is evaluated separately for each age group as well as for the hospital and ICU occupancy, respectively. This results in four estimates $\tilde{\mathbf {r}_\text{ h,1}^*}$, $\tilde{\mathbf {r}_\text{ h,2}^*}$, $\tilde{\mathbf {r}_\text{ icu,1}^*}$ and $\tilde{\mathbf {r}_\text{ icu,2}^*}$ for each country.

The estimated case-specific admission rates obtained from (17) are presented for Austria in Fig. 6.

**Fig. 7:**
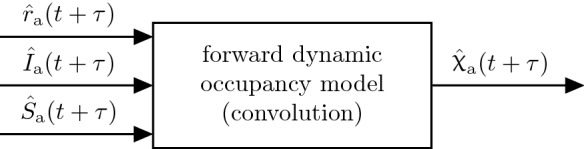
Using the convolution sums in (12), predictions of occupancies are obtained from predicted states of the epidemic (i.e. $\hat{I}_\text {a},\hat{S}_\text {a}$) and projections of case specific admission rates $\hat{r}_{a}$

**Fig. 8:**
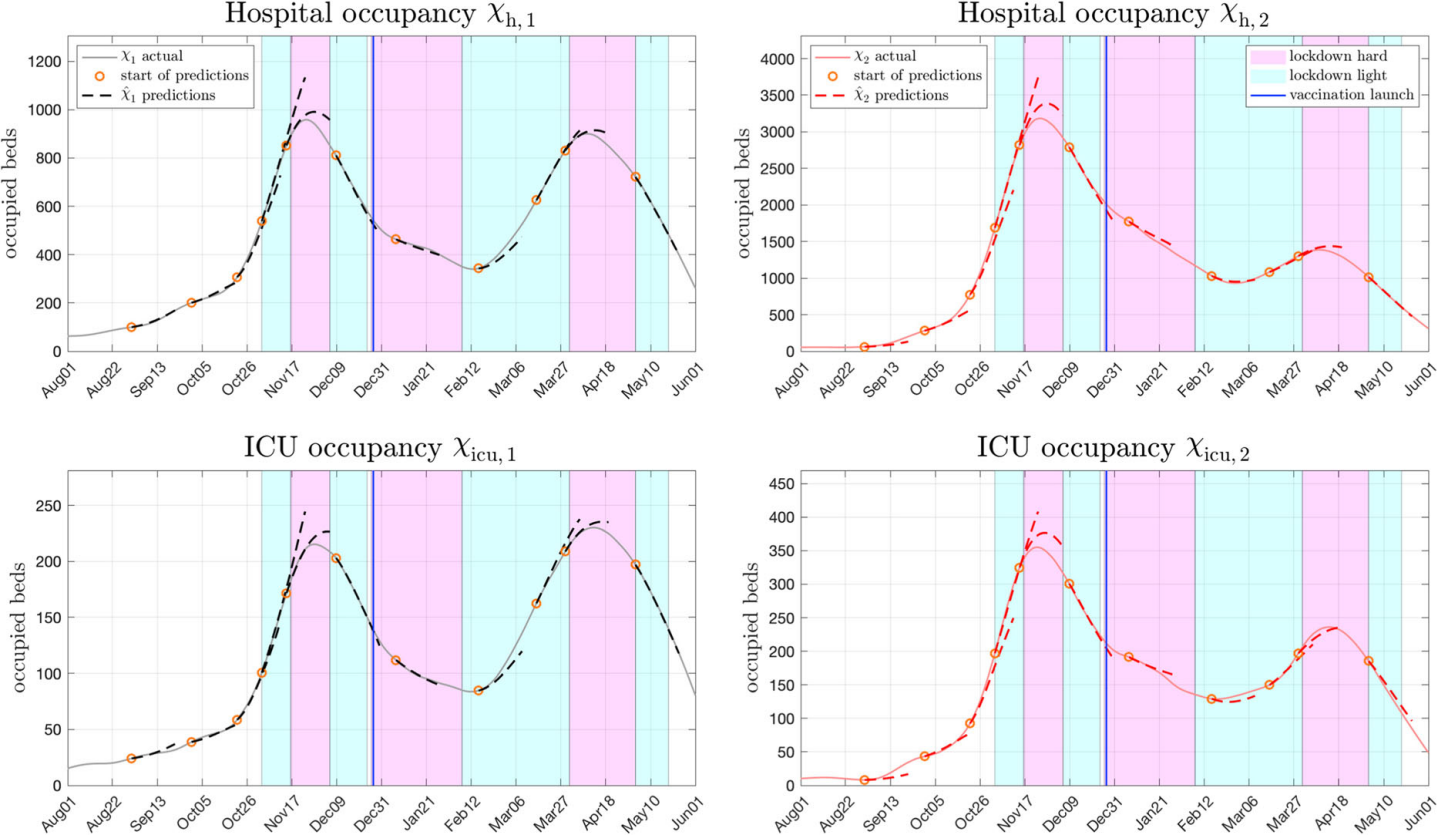
Hospital and ICU occupancy for both age groups in Austria. The corresponding predictions are obtained as shown by the forward dynamic occupancy model presented in Fig. 7. Predictions are represented by dashed lines in the respective colour of the age group with starting points marked as orange circles. Solid lines represent the actual courses

**Fig. 9:**
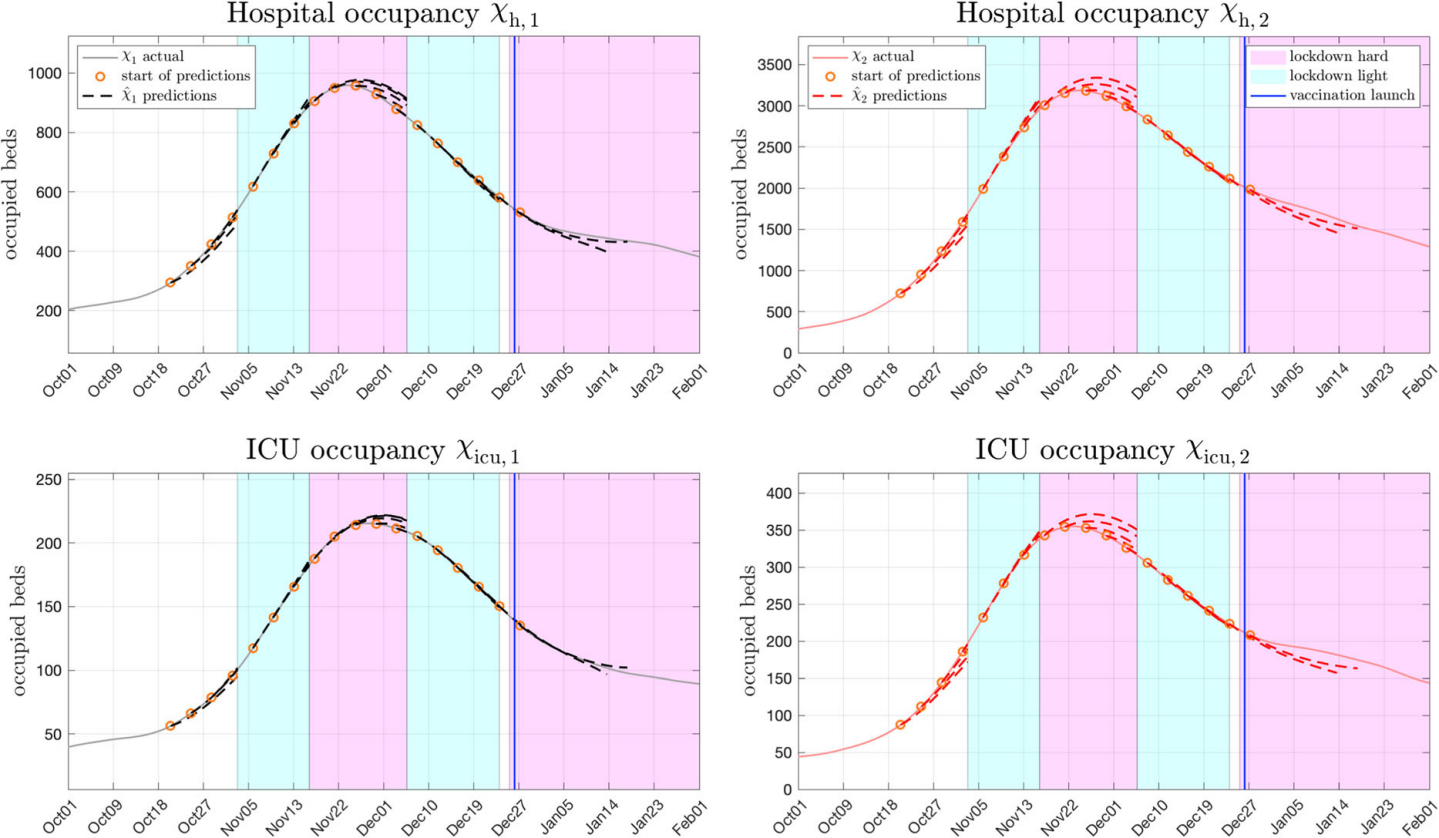
Estimated hospital and ICU occupancy predictions versus reported occupancies during the large fall 2020 Austrian COVID-19 peak in detail. The rolling forecasts starting on October 1st and ending on February 1st, are updated every four days

They are shown as solid lines in all plots. Black lines indicate the younger age group (0-64 yrs.) whereas red lines correspond to the older group (65+ yrs.). It can be seen, that the case-specific hospitalization and ICU admission rates for all age groups vary significantly over time. It can be also clearly seen that the risk of being assigned to hospital or ICU care after an infection differs by roughly one order of magnitude between the age groups. The corresponding results for France and the Netherlands are again given in the Appendix.

## Analysis and forecasts of age-structured hospital and ICU occupancies

The following shows how the occupancies of hospital and ICU beds for different age groups based on the estimated exogenous drivers and the estimated case-specific admission rates can be predicted and how rolling forecasts are obtained. The graphs in Fig. 6 show the *projected* case-specific admission rates $\hat{r}(t+\tau )$ in addition to the estimated admission rates $\tilde{r}^*(t)$. The beginnings of these projections are marked with an orange circle and all projections last three three weeks into the future (i.e. $0<\tau \le 21$) making use of extrapolation by low-order polynomials. Since the projections of $\hat{r}(t+\tau )$ and the epidemic states are required for the occupancy forecasts, the same points in time are chosen as for the projections of $\hat{u}$ in Fig. 4.

Figure 7 schematically shows how the predictions of hospital ${\hat{{\upchi }}}_\text{ h,a }(t+\tau )$ or ICU occupancies ${\hat{{\upchi }}}_\text{ icu,a }(t+\tau )$ are determined.

### Hospitalization and ICU occupancy predictions

Predictions about future hospitalizations and ICU occupancies are obtained from the convolution sums (12) where the upper limit is now set to the respective future day $t+\tau$:

18 $$\begin{aligned} \begin{aligned}&{\hat{{\upchi }}}_\text {h,a}(t+\tau ) = \sum \limits _{\begin{array}{c} \theta =\\ t+\tau -d_\text {max} \end{array}}^{t} \varphi _\text {h,a}(t+\tau -\theta ) \tilde{r}_\text {h,a}(\theta ) \pi _\text {a} (\theta ) \\&\quad + \;\;\, \sum \limits _{\theta =t+1}^{t+\tau } \varphi _\text {h,a}(t+\tau -\theta ) \hat{r}_\text {h,a}(\theta ) \hat{\pi }_\text {a} (\theta ) \\&\quad {\hat{{\upchi }}}_\text {icu,a}(t+\tau ) = \sum \limits _{\begin{array}{c} \theta = \\ t+\tau -d_\text {max} \end{array}}^{t} \varphi _\text {icu,a}(t+\tau -\theta ) \tilde{r}_\text {icu,a}(\theta ) \pi _\text {a} (\theta ) \\&\quad + \;\;\, \sum \limits _{\theta =t+1}^{t+\tau } \varphi _\text {icu,a}(t+\tau -\theta ) \hat{r}_\text {icu,a}(\theta ) \hat{\pi }_\text {a} (\theta ) \\ \end{aligned} \end{aligned}$$

Note that the convolution sums in (18) have to be segregated into two parts each: The first part, which contains only those time arguments $\theta$ which refer to *past* time instants up to the present (i.e. *t*) and the second part, where only *future* time instants are considered. Consequently, the first sum, e.g. for hospitalizations, uses $\tilde{r}_\text {h,a}$ and $\pi _\text {a}$, whereas in the second sum the respective *predictions* $\hat{r}_\text {h,a}$, $\hat{\pi }_\text {a}$ are used. The latter predictions of the production rates (8) are given as

19 $$\begin{aligned} \hat{\pi }_\text {a}(\theta ) = \hat{\lambda }_\text {a}(\theta ) \hat{S}_\text {a}(\theta ), \end{aligned}$$

which in turn make use of the predictions of the epidemic states $\hat{I}_\text {a}(\theta ),\hat{S}_\text {a}(\theta )$, see Fig. 3.

### Analysis of hospitalization and ICU occupancy forecasts

In the case of Austria, the predicted hospital and ICU occupancies for both age groups obtained from (18) are shown in Fig. 8, for France and the Netherlands the reader is referred to the Appendix. In the corresponding Figures, the three week predictions for $\tau =1,2,\ldots ,21$ days ahead are represented by dashed lines in the colour of the respective age group (black, red). For comparison, the actually reported numbers are shown as solid curves. Note that the starting points of the predictions, displayed as orange circles, were again chosen equal to those in Fig. 4 in order to keep context. Naturally, prediction quality is of especial interest during strong epidemic surges as the one that happened in Austria during fall 2020. To shed more light on this particular phase, a more detailed analysis is shown in Fig. 9, where predictions were updated every four days. Following the assumption that the epidemiological course will significantly change with altering governmental interventions, predictions extending into such a turn of events (i.e. lockdowns) are cut off.

### Statistics of occupancy predictions

This subsection presents an analysis and statistics for multi-week forecasts of the age-structured hospital and ICU occupancies for Austria. Corresponding results for France and the Netherlands can be found in the appendix.

**Fig. 10:**
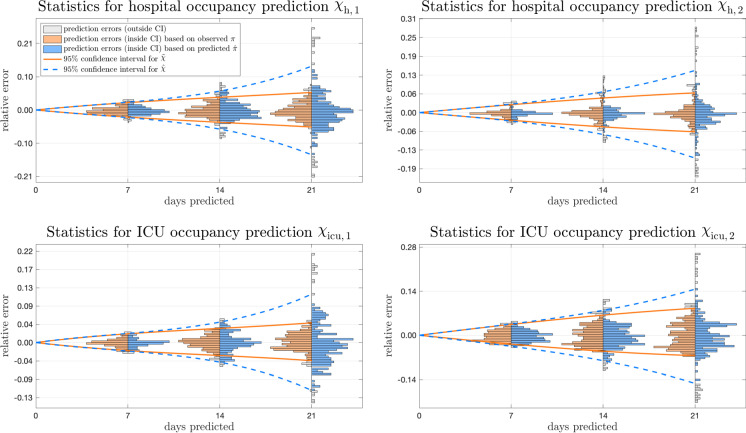
Statistics showing hospital and ICU occupancy predictions for 7, 14 and 21 days for Austria. The relative prediction error with respect to the highest observed historical occupancies is shown in orange for ${\tilde{{\upchi }}}_\text{ h,a }$ and ${\tilde{{\upchi }}}_\text{ icu,a }$ and in blue for ${\hat{{\upchi }}}_\text{ h,a }$ and ${\hat{{\upchi }}}_\text{ icu,a }$

Figure 10 shows the relative errors (with respect to the corresponding highest reported historical occupancy) of hospital and ICU occupancy predictions after 7, 14 and 21 days, respectively, for both age groups. To allow for a statistically meaningful evaluation, predictions were generated/updated on a daily basis from August 1, 2020 to May 11, 2021.

In this analysis, two different variants are considered: First, the predicted occupancies ${\hat{{\upchi }}}_\text{ h,a }(t+\tau )$ and ${\hat{{\upchi }}}_\text{ icu,a }(t+\tau )$ defined by (18) are analysed. In a second variant, in comparison, $\hat{\pi }_\text {a} (\theta )$ in the second sum of (18) was replaced by $\pi _\text {a} (\theta )$ to examine the impact of epidemic forecasts:

20 $$\begin{aligned}&{\tilde{{\upchi }}}_\text {h,a}(t+\tau ) = \sum \limits _{\begin{array}{c} \theta = \\ t+\tau -d_\text {max} \end{array}}^{t} \varphi _\text {h,a}(t+\tau -\theta ) \tilde{r}_\text {h,a}(\theta ) \pi _\text {a} (\theta ) \nonumber \\&\quad + \;\;\, \sum \limits _{\theta =t+1}^{t+\tau } \varphi _\text {h,a}(t+\tau -\theta ) \hat{r}_\text {h,a}(\theta ) \pi _\text {a} (\theta ) \nonumber \\&\quad {\tilde{{\upchi }}}_\text {icu,a}(t+\tau ) = \sum \limits _{\begin{array}{c} \theta = \\ t+\tau -d_\text {max} \end{array}}^{t} \varphi _\text {icu,a}(t+\tau -\theta ) \tilde{r}_\text {icu,a}(\theta ) \pi _\text {a} (\theta ) \nonumber \\&\quad + \;\;\, \sum \limits _{\theta =t+1}^{t+\tau } \varphi _\text {icu,a}(t+\tau -\theta ) \hat{r}_\text {icu,a}(\theta ) \pi _\text {a} (\theta ) \end{aligned}$$

Obviously, the sums in (20) can only be evaluated ex-post, once the epidemic data up until $t+\tau$ are available. Although this variant cannot be used for forecasts in real-time, the retrospective evaluation of ${\tilde{{\upchi }}}_\text{ h,a }(t+\tau )$ and ${\tilde{{\upchi }}}_\text{ icu,a }(t+\tau )$ based on the observed production rate enables the accuracy of the dynamic occupancy model and the admission rate prediction to be quantified. In general, the comparison of the two variants allows to investigate the sensitivity of the predictions to changing case-specific admission rates over the predicted time interval as well as the influence of the projections of future epidemiological development based on the exogenous drivers.

The representation of the prediction errors in normalised histograms (orange bins for ${\tilde{{\upchi }}}_\text{ h,a }$ and ${\tilde{{\upchi }}}_\text{ icu,a }$, blue bins for ${\hat{{\upchi }}}_\text{ h,a }$ and ${\hat{{\upchi }}}_\text{ icu,a }$) in Fig. 10 allows for an analysis of their distribution and provides a means to determine 95% confidence intervals that can be used to assess the accuracy and reliability of future rolling forecasts. While the relative prediction errors after 7 days are small for both, more significant deviations with larger errors for the (true) predictions based on (18) appear after 14 and 21 days, as expected. The higher error bandwidth can be explained by the uncertainty of long-term predictions of the exogenous drivers. The statistics based on (20), i.e. the prediction under the assumption that the future course of the infection numbers is already known, shows that the dynamic occupancy model in combination with the admission rate prediction provides very accurate forecasts over several weeks.

The results of the statistical analysis are also presented in Table 1 in terms of the RMSE and in Table 2 using the normalised RMSE (NRMSE), respectively. In particular, the NRMSE values (again, related to the highest observed historical occupancies) of the prediction errors show that the proposed methodology can produce very accurate predictions of future hospital and ICU occupancies.

**Table 1:**
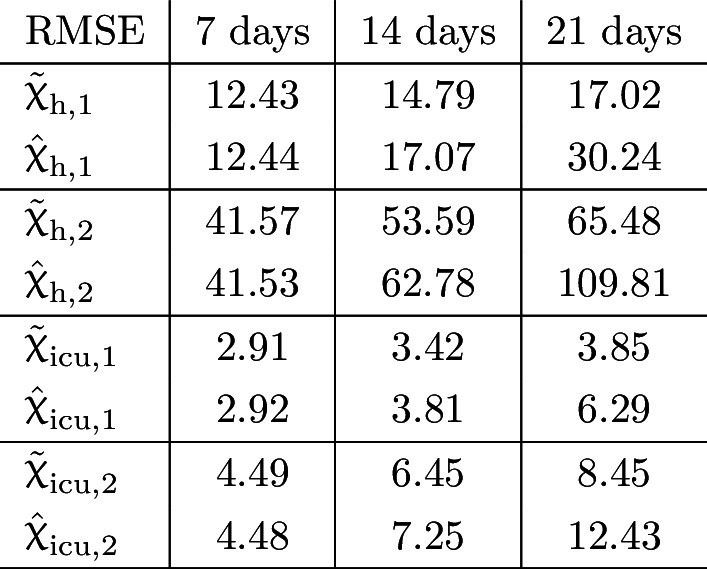
RMSE of the prediction errors at $\tau =7, 14$ and 21 days, respectively, concerning occupied beds for both age groups in Austria

**Table 2:**
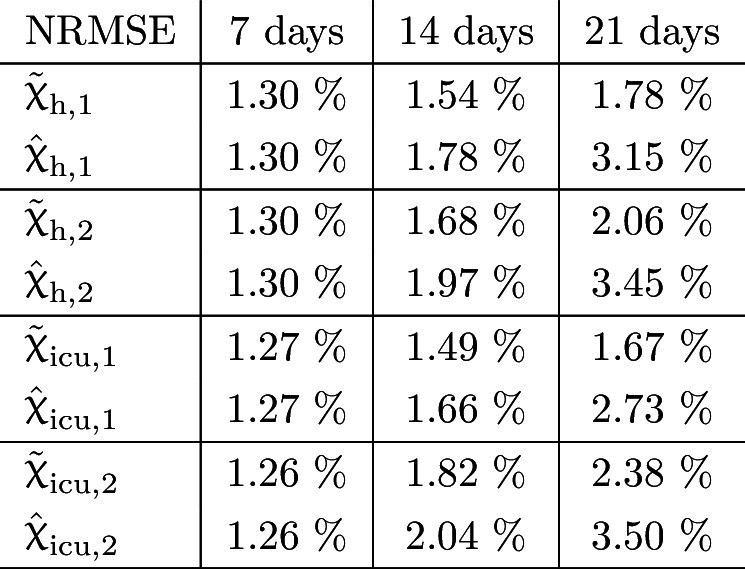
NRMSE of the prediction errors at $\tau =7, 14$ and 21 days, respectively, concerning occupied beds in percent relative to their according historic maximum for both age groups in Austria

## Conclusion

We presented a methodology to quantitatively predict age segregated COVID-19 case numbers and hospitalization and ICU occupancies. This was accomplished by combining an established compartmental model with a methodology from nonlinear control theory and convolution techniques. No prior assumptions on parameter values have to be made as all parameters can be obtained from data in an online fashion. Thus, users of the method are relieved of the task to find suitable parameter sets of the pandemic model or realistic assumptions for predictions. Predictions up to three weeks into the future for case numbers and occupancies are given for several countries and a statistical assessment quantitatively illustrates the accuracy of the method.

For the online prediction of the aggregated external drivers *u*(*t*) simple and robust linear gradient-based functions have been used. Moreover, characteristics and limitations of the course of *u*(*t*) have been derived from historic data.

The methodology and its predictive power could be also used to evaluate the effectiveness of measures such as lockdowns or vaccination campaigns, it allows for an early warning system of oncoming waves, and it enables decision makers to evaluate the pandemic situation by incorporating future developments of both state variables and occupancies.

## Figures and Tables

**Fig. 11:**
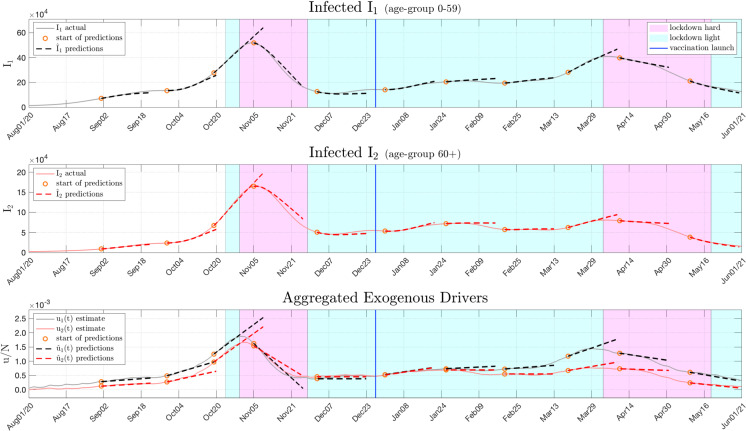
Predicting the course of the disease for France. For different points in time, linear projections $\hat{u}_{1,2}$ (dashed lines) of the aggregated exogenous drivers are used to create forecasts of the number of infected three weeks into the future. The reported number of infected *I* are based on the cases reported by the French Government [38]

**Fig. 12:**
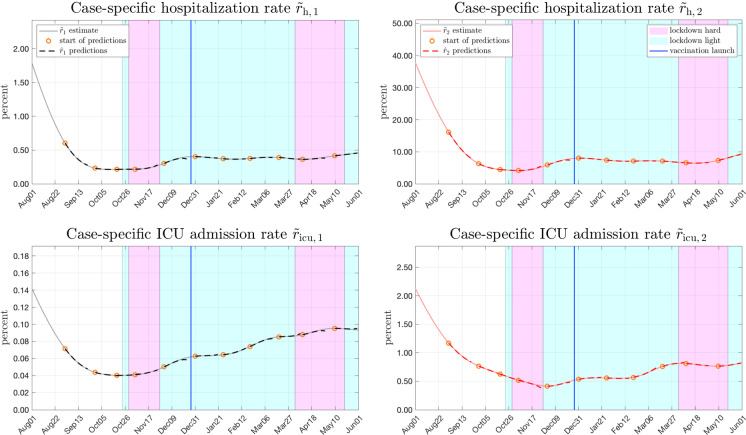
Case-specific hospitalization and ICU admission rates in France for both age groups (solid lines), including rolling forecasts (dashed lines). The forecasts $\hat{r}$ start on the same days as the projections of $\hat{u}$ in Fig. 11

**Fig. 13:**
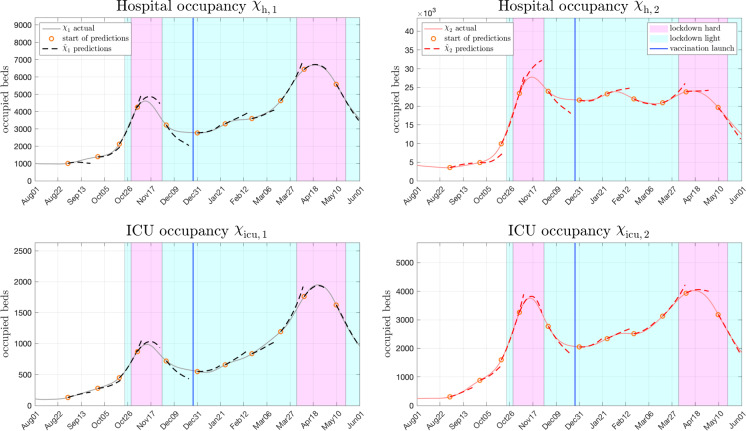
Hospital and ICU occupancy for both age groups in France. Predictions are shown as dashed curves in the respective colour of the age group with starting points marked as orange circles. Solid lines represent the actual courses

**Fig. 14:**
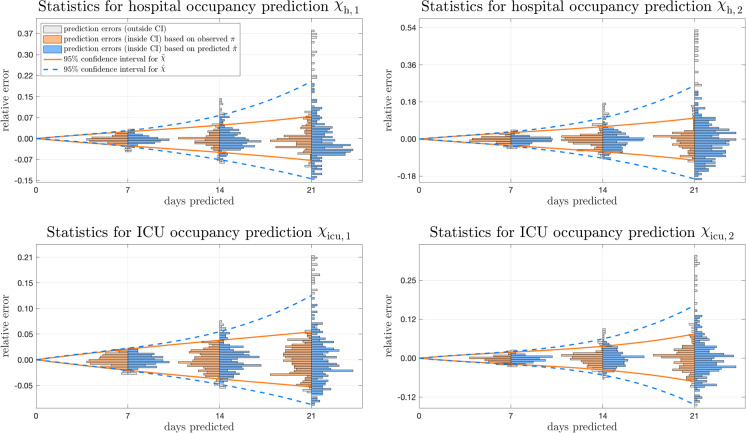
Statistics showing hospital and ICU occupancy predictions for 7, 14 and 21 days for France. The relative prediction error with respect to the highest observed historical occupancies is shown in orange for ${\tilde{{\upchi }}}_\text{ h,a }$ and ${\tilde{{\upchi }}}_\text{ icu,a }$ and in blue for ${\hat{{\upchi }}}_\text{ h,a }$ and ${\hat{{\upchi }}}_\text{ icu,a }$

**Fig. 15:**
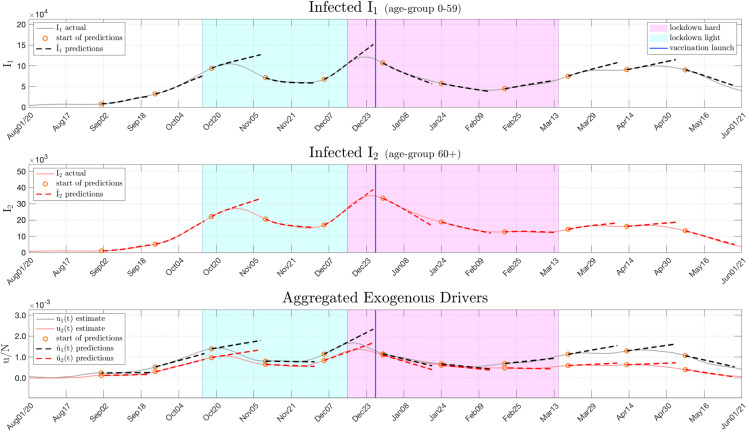
Predicting the course of the disease for the Netherlands. For different points in time, linear projections $\hat{u}_{1,2}$ (dashed lines) of the aggregated exogenous drivers are used to create forecasts of the number of infected three weeks into the future. The reported number of infected *I* are based on the cases reported by the RIVM [39]

**Fig. 16:**
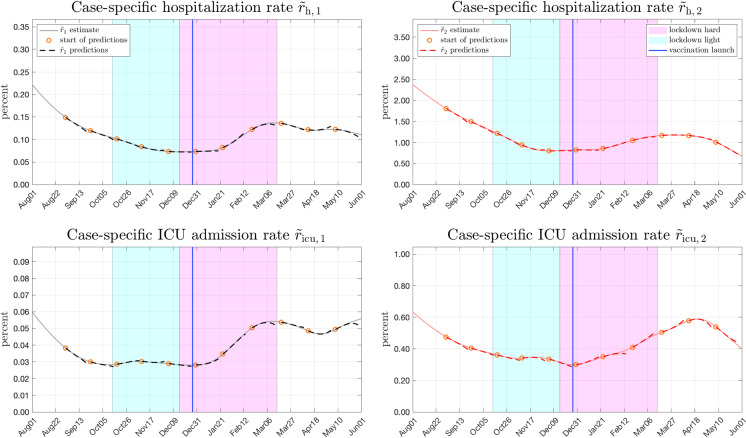
Case-specific hospitalization and ICU admission rates in the Netherlands for both age groups (solid lines), including rolling forecasts (dashed lines). The forecasts $\hat{r}$ start on the same days as the projections of $\hat{u}$ in Fig. 15

**Fig. 17:**
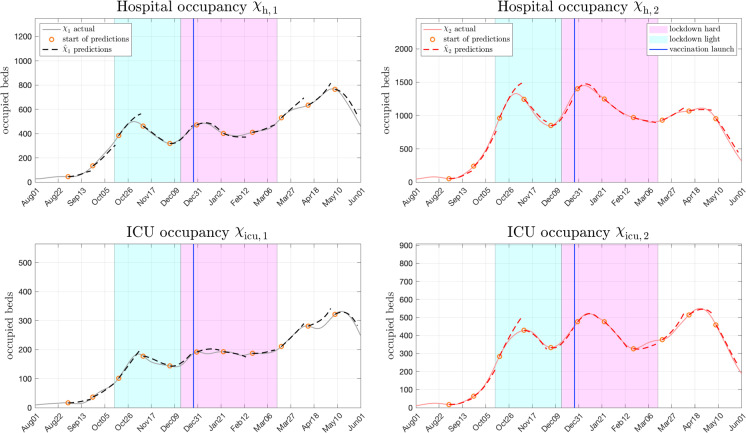
Hospital and ICU occupancy for both age groups in the Netherlands. Predictions are shown as dashed curves in the respective colour of the age group with starting points marked as orange circles. Solid lines represent the actual courses

**Fig. 18:**
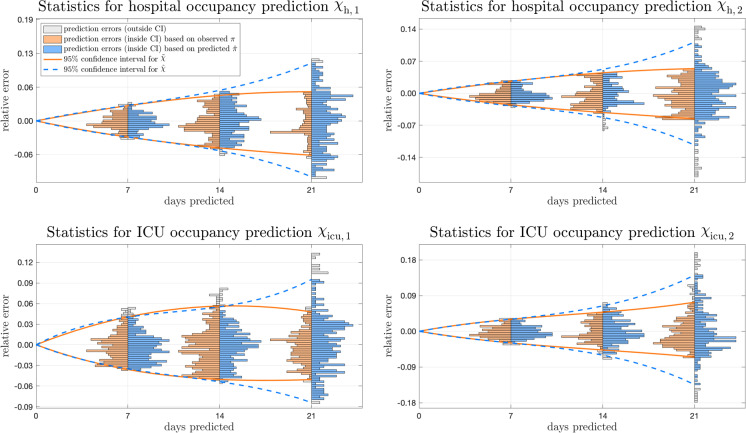
Statistics showing hospital and ICU occupancy predictions for 7, 14 and 21 days for the Netherlands. The relative prediction error with respect to the highest observed historical occupancies is shown in orange for ${\tilde{{\upchi }}}_\text{ h,a }$ and ${\tilde{{\upchi }}}_\text{ icu,a }$ and in blue for ${\hat{{\upchi }}}_\text{ h,a }$ and ${\hat{{\upchi }}}_\text{ icu,a }$

## Data Availability

The data used in this paper is taken from the *Datenplattform COVID-19* in case of Austria [37], from the publicly available COVID-19 database of *data.gouv.fr* in case of France [38] and from *RIVMdata* in case of the Netherlands [39].

## Appendix

### Estimations of different countries

More prediction results for the age-structured model based on the proposed methodology for different countries are presented in the following:

- **Predictions of the course of the disease** for France are shown in Fig. 11 and for the Netherlands in Fig. 15.
- **Case-specific admission rates** for France are shown in Fig. 12 and for the Netherlands in Fig. 16.
- **Occupancy predictions** for France are shown in Fig. 13 and for the Netherlands in Fig. 17.
- **Prediction accuracies** for France are shown in Fig. 14 and for the Netherlands in Fig. 18.

## Variables

*I*: Reported active cases [reported number of currently infected people (positive tested)]
*S*: Susceptibles (people that are susceptible to the COVID-19 disease)
*u*: Aggregated exogenous drivers (leading indicator for the course of the disease obtained from nonlinear control theory)
*N*: Population size (number of people in the considered country or age group)
$\beta$: Transmission rate (rate (speed) describing the spread of COVID-19)
$\gamma$: Recovery rate (rate at which infected individuals recover)
$\pi$: Production rate (daily fow of individuals from compartment S to I, i.e. $\pi$ = $\beta$*SI*/*N* for the standard SIR-model)
*r*: Case-specific admission rate (share of people tested positive with COVID-19 who are admitted to the hospital or ICU)
${\upchi }$: Occupancy (number of people/occupied beds in hospital or ICU at a specific day)
$d_\text{ max }$: Maximal days in hospital or ICU (maximal number of days an individual of an age group occupies a hospital or ICU bed due to COVID-19)
$\varphi (\tau )$: Probability of care (probability for being in care $\tau$ days after infection, conditional to prior hospitalization or ICU assignment)

## Indices

a: Age group (placeholder for age groups)
h: Hospital (refers to hospital)
icu: Intensive care unit (refers to ICU)
1: Younger age group (refers to the younger age group of a country)
2: Older age group (refers to the older age group of a country)

## Footnotes

1 The subscript *a* is omitted as well for a more compact description in the following
